# Improvement in the Mortality-to-Incidence Ratios for Gastric Cancer in Developed Countries With High Health Expenditures

**DOI:** 10.3389/fpubh.2021.713895

**Published:** 2021-08-17

**Authors:** Tzu-Wei Yang, Chi-Chih Wang, Wei-Cheng Hung, Yu-Hsiang Liu, Wen-Wei Sung, Ming-Chang Tsai

**Affiliations:** ^1^Division of Gastroenterology and Hepatology, Department of Internal Medicine, Chung Shan Medical University Hospital, Taichung, Taiwan; ^2^School of Medicine, Chung Shan Medical University, Taichung, Taiwan; ^3^Institute of Medicine, Chung Shan Medical University, Taichung, Taiwan; ^4^Department of Otolaryngology, Chung Shan Medical University Hospital, Taichung, Taiwan; ^5^Department of Medical Education, Chung Shan Medical University Hospital, Taichung, Taiwan; ^6^Department of Urology, Chung Shan Medical University Hospital, Taichung, Taiwan

**Keywords:** gastric cancer, mortality, incidence, mortality-to-incidence ratio, expenditure

## Abstract

The mortality-to-incidence ratio (MIR) is widely used to evaluate the efficacy of cancer management outcomes for individual countries. However, the association among health care expenditure, human development index (HDI), and changes in MIR over time (δMIR) remains unknown. We aimed to elucidate the significance between these indicators and gastric cancer outcomes in different countries. Among the regions, Asia had the highest number of new gastric cancer cases, gastric cancer-related deaths, age-standardized ratio of incidence, and mortality. Chile had the highest age-standardized ratio (ASR) for gastric cancer incidence and the highest ASR for mortality. Moreover, MIR was highest in Africa (0.91) and lowest in North America (0.43). Of note, MIR was negatively associated with HDI, current health expenditure (CHE) per capita, and CHE/GDP % and δMIR was positively associated with CHE/GDP % in countries with very high HDI. However, δMIR showed no significant associations with these indicators in the countries analyzed. In conclusion, increased HDI, CHE per capita, and CHE/GDP are associated with improved gastric cancer outcomes. In addition, the δMIR could be an indicator that can be used to evaluate the improvement in cancer management outcomes over time.

## Introduction

Along with increasing life expectancy, cancer has become the leading cause of death in humans, and the incidence of cancer and cancer-related deaths is growing rapidly (1). Gastric cancer is one of the most common cancers worldwide; gastric cancer has the fifth highest incidence among all cancers, and the third leading cause of cancer-related deaths in 2018 (2). The epidemiology of gastric cancer varies in different regions. For instance, gastric cancer incidence and mortality rates are highest in Eastern Asia, while the rates in North America are lower than those in other regions (2, 3).

Several risk factors are involved in gastric cancer, such as obesity and gastro-esophageal reflux disease, which are risk factors for cardia gastric cancer, whereas Helicobacter pylori (HP) infection, alcohol consumption, and intake of salty and salt-preserved food are risk factors for non-cardia gastric cancer (4–6). Efforts were made to improve the incidence rate of gastric cancer, such as HP infection eradication, food preservation, and lifestyle modifications (3, 6). In addition, the improvements in population-based screening involving biomarkers and early endoscopy-based detection, as well as the improvements in the treatment modalities for gastric cancer, had remarkably reduced the mortality rate of gastric cancer. However, the mortality rates vary in different regions (7–9). Hence, a good indicator to evaluate the improvement of gastric cancer management and outcome is needed.

The mortality-to-incidence ratio (MIR) is an indicator that is widely used to evaluate cancer management outcomes in different countries (10–12). MIR was found to be associated with the success of cancer screening, surveillance, and treatment in colorectal cancer and in other cancer types worldwide (10–12). MIRs were reported to be lower in countries with advanced public health systems (11–13). Nonetheless, there is a lack of indicators to evaluate the improvement of gastric cancer management outcomes over time and the association with health expenditure among countries.

To elucidate the relationship, we conducted an analysis using the 2012 and 2018 GLOBOCAN gastric cancer incidence and mortality statistics from 59 countries to determine the association among the human development index (HDI), current health expenditure (CHE), MIR, and the change in MIR between 2012 and 2018.

## Methods

Cancer epidemiological data were obtained from the GLOBOCAN project (http://gco.iarc.fr/), which is maintained by the International Agency for Research on Cancer of the World Health Organization. GLOBOCAN, which received an update in 2018, is a public access database that provides contemporary estimates of cancer epidemiology worldwide. HDI data was obtained from the Human Development Report Office of the United Nations Development Programme (http://hdr.undp.org/en). HDI was categorized as very high (0.800–1.000), high (0.700–0.799), medium (0.550–0.699), and low (0.350–0.549). In this investigation, countries were excluded based on the data quality report in GLOBOCAN (*N* = 123), based on missing data (*N* = 7), and based on outlier MIRs (*N* = 2). A total of 59 countries were included in the final analysis. No personal information was used in this study, and all data were obtained from public databases. No approval of the Institutional Review Board was needed as well.

Data on health expenditures, including the per capita CHE and the CHE/GDP (ratio of CHE to the percentage of gross domestic product or GDP), were obtained from the World Health Statistics database (https://www.who.int/gho/publications/world_health_statistics/en/). MIR was defined as the ratio of the crude rate (CR) of mortality to the CR of incidence, as previously described (10, 14–16). The δMIR was defined as the difference between the MIR in 2012 and in 2018 (δMIR = MIR [in 2012]–MIR [in 2018]) (17). Associations among the MIR, δMIR, and other factors in various countries were estimated using Spearman's rank correlation coefficient using the SPSS statistical software version 15.0 (SPSS, Inc., Chicago, IL). p values of <0.05 were considered statistically significant. Scatterplots were generated using Microsoft Excel.

## Results

### Epidemiology of Gastric Cancer According to Regions

To evaluate the global burden of gastric cancer in 2018, we analyzed the global data and summarized them in Table 1. Asia had the highest numbers of new cases (715,905) and deaths (532,673), as well as the highest age-standardized rate (ASR) of new cases (13.4) and deaths (9.9), followed by Latin America and the Caribbean (9.1 and 6.1, respectively). In contrast, the lowest numbers of new cases (25,602) and deaths (11,060) were found in North America, which also had the lowest ASR of new cases (3.9) and deaths (1.7). Of note, the MIR was highest in Africa, with a value of 0.91, and lowest in North America, with a value of 0.43.

**Table 1:** Summary of the number, crude rank, age-standardized rate, and mortality-to-incidence ratio of gastric cancer according to the regions.

	**New cases**			**Deaths**			
**Region**	**Number**	**CR**	**ASR**	**Number**	**CR**	**ASR**	**MIR**
Africa	30,134	2.3	3.9	27,611	2.1	3.7	0.91
Asia	715,905	15.9	13.4	532,673	11.8	9.9	0.74
Europe	117,855	16.2	7.7	86,724	11.9	5.5	0.73
Latin America and the Caribbean	60,170	9.3	8.1	45,568	7.0	6.1	0.75
North America	25,602	7.2	3.9	11,060	3.1	1.7	0.43
Oceania	2,915	7.2	4.7	1,770	4.4	2.8	0.61

### Epidemiology and the Three Indicators of Gastric Cancer in Different Countries

The HDI, CHE, cancer incidence, cancer mortality, and MIR in gastric cancer in 59 countries in 2018 are summarized in Table 2. Chile had the highest ASR for gastric cancer incidence and the highest ASR for mortality. The United States of America had the lowest MIR of 0.42, whereas Cyprus had the highest MIR of 0.92. By comparing the δMIRs of the investigated countries, we found that the MIRs in most countries decreased in 2018. Fiji had the highest δMIR of 0.30, followed by Belarus and Chile, with a δMIR of 0.26.

**Table 2:** Summary of human development index, current health expenditure, cancer incidence, cancer mortality, and mortality-to-incidence ratio in gastric cancer of selected countries (*N* = 59).

		**Current health expenditure**		**Incidence**			**Mortality**			**Mortality-to-incidence ratio**		
**Country**	**HDI**	**Per capita**	**% of GDP**	**Number**	**CR**	**ASR**	**Number**	**CR**	**ASR**	**2012**	**2018**	**δMIR**
Argentina	0.830	998	6.8	3,555	8.1	6.1	2,790	6.3	4.6	0.88	0.78	0.10
Australia	0.938	4,934	9.4	1,949	8.0	4.3	952	3.9	2.0	0.56	0.49	0.07
Austria	0.914	4,536	10.3	983	11.5	4.9	608	7.1	2.9	0.65	0.62	0.03
Bahrain	0.838	1,190	5.2	30	1.9	3.0	27	1.7	2.9	0.71	0.90	−0.19
Belarus	0.817	352	6.1	2,705	29.1	16.0	1,582	17.0	9.2	0.84	0.58	0.26
Belgium	0.919	4,228	10.5	1,303	11.7	5.5	577	5.2	2.3	0.68	0.44	0.24
Brazil	0.761	780	8.9	19,198	9.2	7.5	14,288	6.8	5.5	0.82	0.74	0.08
Bulgaria	0.816	572	8.2	1,214	17.6	7.6	954	13.8	5.8	0.81	0.78	0.03
Canada	0.922	4,508	10.4	2,781	7.7	3.8	1,549	4.3	2.0	0.58	0.56	0.02
Chile	0.847	1,102	8.1	4,514	25.1	16.5	2,933	16.3	10.4	0.91	0.65	0.26
Colombia	0.761	374	6.2	6,783	13.8	11.9	4,920	10.0	8.5	0.85	0.73	0.12
Costa Rica	0.794	929	8.1	803	16.4	12.3	605	12.3	8.9	0.70	0.75	−0.05
Croatia	0.837	852	7.4	754	18.6	7.9	637	15.7	6.3	0.81	0.84	−0.03
Cuba	0.778	826	10.9	1,163	10.3	5.4	809	7.2	3.7	0.81	0.70	0.11
Cyprus	0.873	1,563	6.8	107	9.1	5.5	98	8.4	4.6	0.77	0.92	−0.15
Czechia	0.891	1,284	7.3	1,241	11.9	5.5	832	8.0	3.5	0.69	0.67	0.02
Denmark	0.930	5,497	10.3	489	8.7	4.0	345	6.1	2.7	0.56	0.70	−0.14
Ecuador	0.758	530	8.5	2,171	13.0	12.1	1,675	10.0	9.2	0.94	0.77	0.17
Egypt	0.700	157	4.2	2,285	2.3	2.8	1,806	1.8	2.2	0.90	0.78	0.12
Estonia	0.882	1,112	6.5	294	23.1	10.9	231	18.1	8.1	0.77	0.78	−0.01
Fiji	0.724	175	3.6	33	3.6	3.6	25	2.7	2.7	1.05	0.75	0.30
Finland	0.925	4,005	9.4	486	9.0	3.8	346	6.4	2.6	0.75	0.71	0.04
France	0.891	4,026	11.1	6,260	9.9	4.6	3,924	6.2	2.8	0.68	0.63	0.05
Germany	0.939	4,592	11.2	12,322	15.4	6.4	7,493	9.4	3.4	0.61	0.61	0.00
Iceland	0.938	4,375	8.6	21	6.3	3.2	15	4.5	2.2	0.65	0.71	−0.06
Ireland	0.942	4,757	7.8	601	12.7	7.0	277	5.9	3.1	0.67	0.47	0.20
Israel	0.906	2,756	7.4	565	6.8	4.8	414	5.0	3.5	0.66	0.74	−0.08
Italy	0.883	2,700	9.0	10,338	18.1	6.6	7,007	12.3	4.2	0.77	0.68	0.09
Jamaica	0.726	294	5.9	285	10.0	8.0	202	7.1	5.5	0.86	0.71	0.15
Kuwait	0.808	1,169	4.0	72	1.7	2.4	64	1.5	2.3	0.64	0.88	−0.24
Latvia	0.854	784	5.8	510	27.1	12.4	390	20.7	8.9	0.76	0.76	0.00
Lithuania	0.869	923	6.5	763	27.2	12.7	617	22.0	9.9	0.77	0.81	−0.04
Luxembourg	0.909	6,236	6.0	53	9.2	4.9	29	5.0	2.6	0.48	0.54	−0.06
Malaysia	0.804	386	4.0	1,507	4.7	4.8	1,192	3.7	3.7	0.46	0.79	−0.33
Malta	0.885	2,304	9.6	56	13.2	5.6	41	9.7	3.8	0.49	0.74	−0.25
Mauritius	0.796	506	5.5	121	9.6	6.2	86	6.8	4.3	0.92	0.71	0.21
Netherlands	0.934	4,746	10.7	1,604	9.6	4.3	1,087	6.5	2.7	0.71	0.68	0.03
New Zealand	0.921	3,554	9.3	349	7.5	4.2	256	5.5	2.9	0.61	0.73	−0.12
Norway	0.954	7,464	10.0	401	7.7	3.8	247	4.7	2.2	0.66	0.61	0.05
Oman	0.834	636	3.8	207	4.3	8.2	175	3.6	7.2	0.85	0.84	0.01
Philippines	0.712	127	4.4	2,904	2.7	3.4	2,379	2.2	2.8	0.84	0.82	0.02
Poland	0.872	797	6.3	5,886	15.8	7.9	5,007	13.4	6.5	0.86	0.85	0.01
Portugal	0.850	1,722	9.0	2,421	24.3	10.3	1,811	18.2	7.2	0.76	0.75	0.01
Qatar	0.848	2,030	3.1	42	1.6	3.9	39	1.4	3.9	0.80	0.88	−0.08
Russian Federation	0.824	524	5.6	33,250	23.4	12.9	27,322	19.3	10.3	0.86	0.83	0.03
Serbia	0.799	491	9.4	1,170	13.6	7.0	929	10.8	5.3	0.82	0.79	0.03
Singapore	0.935	2,280	4.3	845	14.8	7.5	601	10.5	5.4	0.67	0.71	−0.04
Slovakia	0.857	1,108	6.9	1,036	19.3	10.0	585	10.9	5.5	0.71	0.57	0.14
Slovenia	0.902	1,772	8.5	390	19.2	8.4	250	12.3	4.9	0.72	0.64	0.08
South Africa	0.705	471	8.2	1,838	3.2	3.8	1,469	2.6	3.0	0.75	0.81	−0.06
Spain	0.893	2,354	9.2	6,252	13.9	6.2	4,201	9.4	3.9	0.69	0.68	0.01
Sweden	0.937	5,600	11.0	665	6.8	3.1	440	4.5	1.9	0.79	0.66	0.13
Switzerland	0.946	9,818	12.1	895	10.8	5.2	508	6.1	2.7	0.72	0.57	0.15
Thailand	0.765	217	3.8	3,685	5.4	3.4	3,166	4.6	3.0	0.80	0.85	−0.05
Trinidad and Tobago	0.799	1,146	6.0	79	5.8	3.9	59	4.3	2.9	0.78	0.74	0.04
Ukraine	0.750	125	6.1	9,077	20.9	10.9	7,928	18.3	9.4	0.81	0.88	−0.07
United Kingdom	0.920	4,356	9.9	5,100	7.9	3.5	3,329	5.1	2.2	0.68	0.65	0.03
United States of America	0.920	9,536	16.8	22,816	7.1	3.9	9,511	3.0	1.6	0.55	0.42	0.13
Uruguay	0.808	1,281	9.2	426	12.6	7.6	376	11.1	6.5	0.89	0.88	0.01

Next, we analyzed the association of three indicators, namely, HDI, CHE per capita (in USD), and CHE as a percentage of GDP (CHE/GDP, %), with the crude rate of incidence and mortality in gastric cancer (Supplementary Figures 1A–F). However, no association was found among those indicators with the CR of incidence and mortality.

### Association Between MIR and the Three Indicators in Different Countries

**Figure 1:**
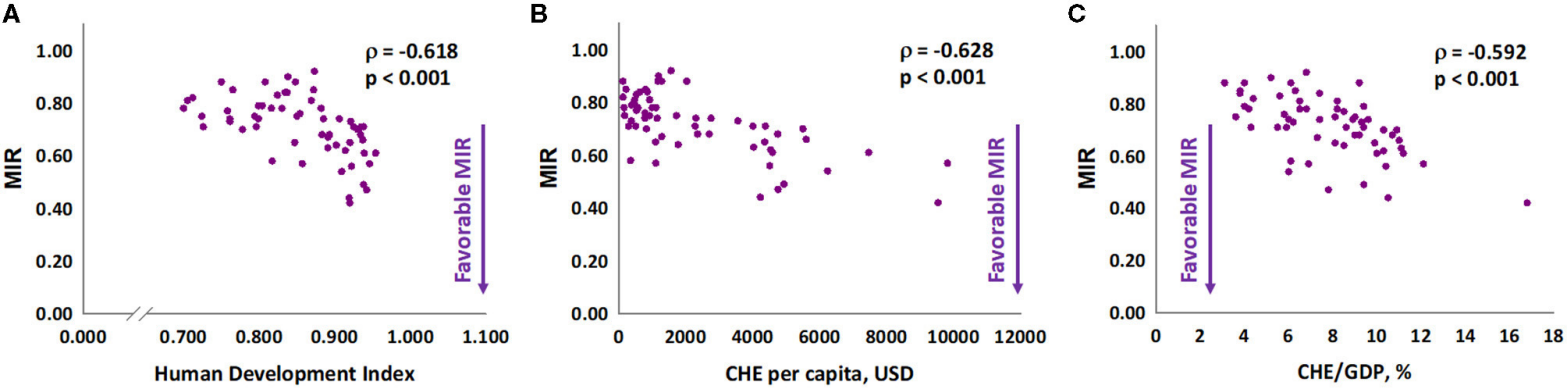
The **(A)** human development index, **(B)** current health expenditure per capita, and **(C)** current health expenditure as a percentage of gross domestic product are significantly associated with the mortality-to-incidence ratio in gastric cancer.

**Figure 2:**
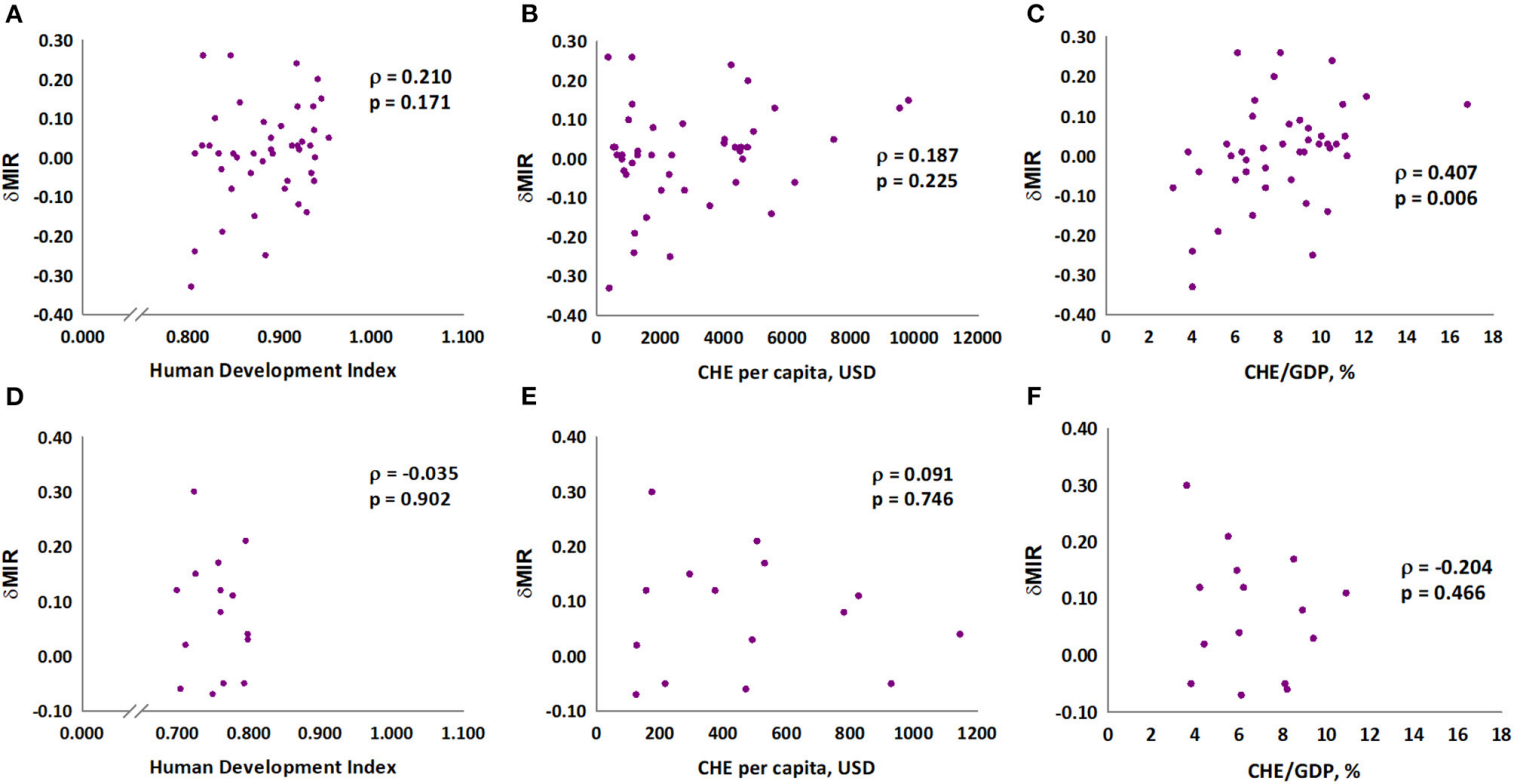
Association between human development index (HDI), current health expenditure per capita, current health expenditure as percentage of gross domestic product and delta mortality-to-incidence ratio in gastric cancer in very high HDI countries **(A–C)** and others **(D–F)**.

We further analyzed the association of MIR with HDI, CHE per capita, and CHE/GDP. These three indicators were negatively associated with the MIR for gastric cancer in 2018 (Spearman's rank correlation coefficient ρ, −0.618, −0.628, and −0.592, respectively; all P < 0.001; Figures 1A–C). Furthermore, we analyzed the association of these three indicators with δMIR from 2012 to 2018, and no significant associations of these indicators with δMIR were found (Supplementary Figure 2). Subgroup analysis showed a positive correlation between δMIR and CHE_GDP in “very high” HDI countries (HDI 0.8–1.0, defined by the United Nations Development Programme) (Spearman's rank correlation coefficient ρ = 0.407, *P* = 0.006; Figure 2).

## Discussion

This study aimed to analyze the association among the levels of economic development, health expenditure, gastric cancer outcomes globally in 2018, and the improvement of gastric cancer management over time. We found that gastric cancer incidence, mortality, rank of all cancers, and cumulative risk of incidence were highest in Asia. Chile had the highest ASR for gastric cancer incidence and the highest ASR for mortality. Overall, our findings suggested that gastric cancer in countries with higher CHE per capita and CHE/GDP ratio had better clinical outcomes. Improvement of gastric cancer management was found in highly developed countries with high health expenditures.

Previous studies have indicated that the MIRs are directly correlated to the health system ranking in lung, colorectal, prostate, stomach, and breast cancers (12). MIRs are also good indicators for evaluating cancer screening and care in colon cancer (10). Rezaeian et al. (18) found that countries with a low HDI index had higher MIRs for all cancers. It was concluded that patterns of cancer occurrence correlate with care disparities at the country level. In comparison, our study revealed that the MIR values were lower in countries with higher health expenditures, especially in countries with higher CHE/GDP ratios. The consistent results suggest a better outcome for patients with gastric cancer in these countries with high health expenditures. Nonetheless, the positive association between δMIR and CHE/GDP ratio in highly developed countries indicates that the improvement of gastric cancer was only made in countries with prosperous economies. Moreover, it may not be long enough to show the advancement in cancer screening, treatment, and health care utilization in different countries in a 6-year interval.

Although the MIR indicates the quality of gastric cancer care, the disparity in gastric cancer incidence and mortality is influenced by many factors. The incidence of gastric cancer is highly dependent on diet, lifestyle, and HP infection in different regions. Japan is one of the regions where gastric cancer is endemic (6). The improvements in clinical outcomes in Japan over the last decades, as well as the improvements in Korea and in other Asian countries, were attributed to their screening program (9, 19, 20). In countries with lower MIR for gastric cancer, the screening modalities and screening program improved with increased medical expenditure. Several novel screening modalities and protocols, such as the use of serum biomarkers, detection of tumor-specific mutations in DNA and proteins in the circulation, and the combination of serum pepsinogen level and esophagogastroduodenoscopy (EGD), improved the early detection of gastric cancer (19, 21–23). In addition, more frequent screening via either EGD or radiography has been reported to improve the early detection and mortality of gastric cancer (7, 19). However, the frequency of screening may be associated with increased medical expenditures in some developed countries.

Medical expenditure also increased with the progression of gastric cancer treatment. The treatment for gastric cancer nowadays includes endoscopic resection, surgical resection, adjuvant chemotherapy, and targeted therapy (3). A recent prospective study revealed the molecular level of gastric cancer, which was a powerful breakthrough for novel target therapy (24). New drugs and treatment strategies, such as adjuvant chemotherapy with S-1, oral fluoropyrimidine in patients with advanced gastric cancer after surgical intervention, and targeted therapy with trastuzumab in HER2-positive advanced gastric cancer patients, have shown survival benefits (25, 26). Advances in treatment strategies developed in recent years have improved gastric cancer mortality, especially in developed countries (24, 27). However, improvements in gastric cancer treatment and outcomes are associated with increased medical expenditure, which is consistent with our findings.

In our study, several countries had negative δMIR values, which may indicate poor clinical outcomes and worsening cancer care. Increased risk of mortality and more postoperative complications were found in some low/middle-income countries. These findings may suggest the need to enhance surgical techniques and postoperative care. Meanwhile, δMIR could be a useful indicator to evaluate the improvement of gastric cancer care in countries with good healthcare systems.

Our study has some limitations. First, not all countries were analyzed in our study, which may not be exactly the overall burden of gastric cancer in the world. Some countries with high gastric cancer incidence, such as Japan, South Korea, and China, were not included in the analysis due to limited data sources. However, we believe that 59 countries could be a representative study of the cost and outcome of the global burden of gastric cancer. Second, we determined the δMIR between 2012 and 2018, and this parameter could serve as an indicator of the improvement in cancer management for individual nations. However, it cannot be a good surrogate for cancer survival because long-term survival is unknown (28). Third, we found that the HDI, CHE per capita, and CHE/GDP were negatively associated with MIR; however, the positive association between δMIR and health expenditure is limited to countries with very high HDI. The unexpected results could be influenced by the cost of screening, treatment, and health care utilization in different countries. Health expenditure and HDI are indicators aimed at targeting several types of outcomes in health and well-being, not only cancer. The correlation between these indicators is indirect and not well-founded. The association was analyzed across countries; hence, the result might not be directly applicable in any single country. Fourth, per capita expenditure in US dollars is not comparable across a diverse group of countries.

In conclusion, we found that δMIR of gastric cancer is positively associated with health care expenditure in very high HDI developed countries. This is the first study to use δMIR as an indicator to evaluate the improvement of gastric cancer management. Future studies are warranted to determine the impact of the health care system on the clinical outcomes of gastric cancer in different countries.

## Data Availability Statement

The original contributions presented in the study are included in the article/Supplementary Material, further inquiries can be directed to the corresponding author/s.

## Author Contributions

T-WY and C-CW: conceptualization. C-CW: methodology and investigation. W-WS: software and formal analysis and project administration. W-WS, Y-HL, and M-CT: validation. T-WY: resources. W-CH: data curation. Y-HL: writing—original draft preparation. W-WS and T-WY: writing—review and editing. C-CW and W-CH: visualization. T-WY: supervision. All authors have read and agreed to the published version of the manuscript.

## Conflict of Interest

The authors declare that the research was conducted in the absence of any commercial or financial relationships that could be construed as a potential conflict of interest. The reviewer XL declared a shared affiliation with the authors, to the handling editor at time of review.

## Publisher's Note

All claims expressed in this article are solely those of the authors and do not necessarily represent those of their affiliated organizations, or those of the publisher, the editors and the reviewers. Any product that may be evaluated in this article, or claim that may be made by its manufacturer, is not guaranteed or endorsed by the publisher.

## Supplementary Material

Supplementary Figure 1 Association between human development index, current health expenditure and the crude rates of **(A,C,E)** incidence, and **(B,D,F)** mortality in gastric cancer.

Supplementary Figure 2 The **(A)** human development index, **(B)** current health expenditure per capita, and **(C)** current health expenditure as a percentage of gross domestic product are not significantly associated with delta mortality-to-incidence ratio in gastric cancer.
