# Supplementary figures and images for: Improvement in the Mortality-to-Incidence Ratios for Gastric Cancer in Developed Countries With High Health Expenditures
Source: Front Public Health. 2021 Aug 17;9:713895. doi: 10.3389/fpubh.2021.713895 (PMC8415830; doi:10.3389/fpubh.2021.713895)

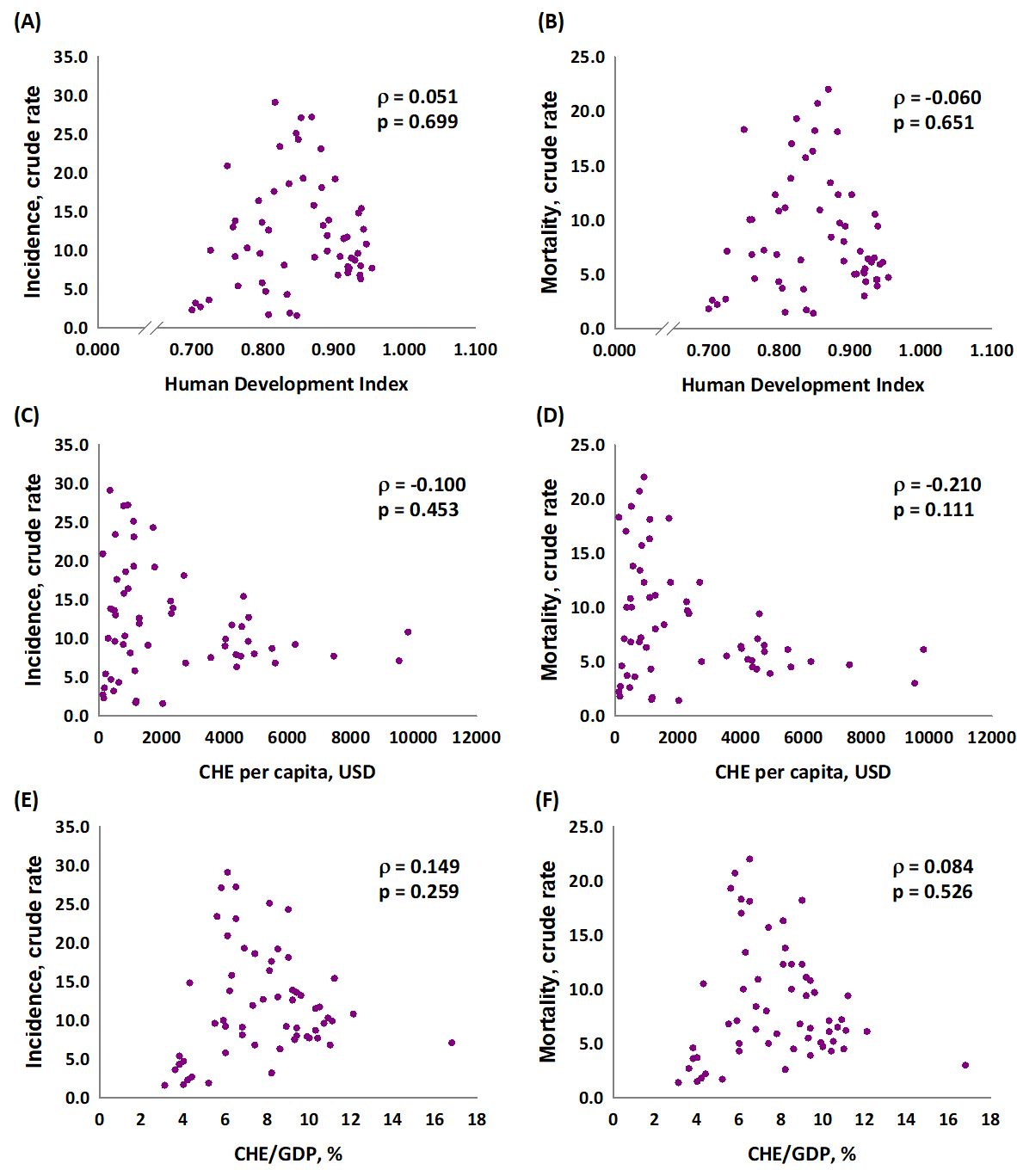

Supplement: Supplementary Figure 1 — Association between human development index, current health expenditure and the crude rates of (A,C,E) incidence, and (B,D,F) mortality in gastric cancer. [file Image_1.TIF]

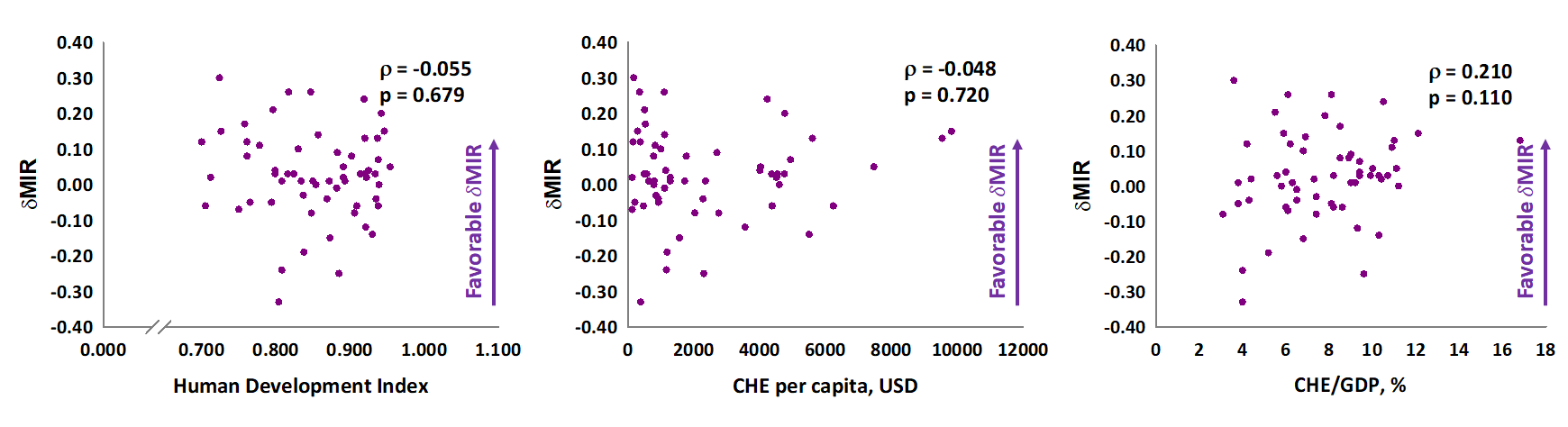

Supplement: Supplementary Figure 2 — The (A) human development index, (B) current health expenditure per capita, and (C) current health expenditure as a percentage of gross domestic product are not significantly associated with delta mortality-to-incidence ratio in gastric cancer. [file Image_2.TIF]
